# Macrophages Can Drive Sympathetic Excitability in the Early Stages of Hypertension

**DOI:** 10.3389/fcvm.2021.807904

**Published:** 2022-01-27

**Authors:** Oliver C. Neely, Ana I. Domingos, David J. Paterson

**Affiliations:** Department of Physiology, Anatomy and Genetics, University of Oxford, Oxford, United Kingdom

**Keywords:** hypertension, dysautonomia, macrophages, SHR, calcium imaging, flow cytometry, stellate neurons

## Abstract

Hypertension is a major health burden worldwide with many cases resistant to current treatments. Hyperactivity of the sympathetic nervous contributes to the etiology and progression of the disease, where emerging evidence suggests that inflammation may underpin the development of sympathetic dysautonomia. This study examined whether macrophages could drive the sympathetic phenotype in Spontaneously Hypertensive Rats (SHR) before animals develop high pressure. Stellate neurons from wild-type control Wistar rats and SHRs were co-cultured with blood leukocytes from their own strain, and also crossed cultured between strains. The calcium transient response to nicotinic stimulation was recorded using Fura-2 calcium imaging, where SHR neurons had a greater calcium transient compared with Wistar neurons. However, when co-cultured with leukocytes, Wistar neurons began to phenocopy the SHR sympathetic hyperactivity, while the SHR neurons themselves were unaltered. Resident leukocyte populations of the SHR and Wistar stellate ganglia were then compared using flow cytometry, where there was a shift in monocyte-macrophage subset proportions. While classical monocyte-macrophages were predominant in the Wistar, there were relatively more of the non-classical subset in the SHR, which have been implicated in pro-inflammatory roles in a number of diseases. When bone marrow-derived macrophages (BMDMs) were co-cultured with stellate neurons, they made Wistar neurons recapitulate the SHR nicotinic stimulated calcium transient. Wistar BMDMs however, had no effect on SHR neurons, even though SHR BMDMs increased SHR neuron responsiveness further above their hyper-responsive state. Taken together, these findings show that macrophages can be potent enhancers of sympathetic neuronal calcium responsiveness, and thus could conceivably play a role in peripheral sympathetic hyperactivity observed in the early stages of hypertension.

## Introduction

A key pathophysiological feature of essential hypertension involves sympathetic hyperactivity (1–5) and impaired vagal parasympathetic tone (2). The sympathetic component of this is by far the best studied and is observed in all stages of essential hypertension (6–9), moreover it can precede hypertension itself (10–12). Persistent heightened sympathetic tone leads to a range of other hypertensive co-morbidities, including cardiac hypertrophy (13, 14), arrhythmia (15), vascular dysfunction (16), insulin resistance (17) and inflammation (18).

Inflammation has emerged as a key pathological feature of essential hypertension (19). Crucially, a simple bone marrow transplant from SHR to Wistar rats increases blood pressure, while the converse decreases it (20), implying the SHR's immune system as a causative factor of the blood pressure phenotype. Clinically, higher levels of C-reactive protein (a common marker of systemic inflammation) (21–24) or IL-6 (an inflammatory cytokine released along with CRP) (23, 24) strongly predict future development of hypertension. This suggests that both inflammation and sympathetic hyperexcitability are co-features of a “pre-hypertensive” state, before any overt cardiovascular disease is evident. Although it has not been established whether inflammation itself is a key driver of the sympathetic phenotype.

There is evidence that the hypertensive inflammatory phenotype may interact with the sympathetic nervous system, in a similar way to that in which immune cells interact with sympathetic neurons in adipose tissue in obesity (25), this being a co-feature of hypertension in the metabolic syndrome. In the central nervous system inflammation can raise sympathetic outflow, which can experimentally induce hypertension in the rat (26). Inhibition of microglial activation with minocycline can stop this (26), and is associated with reduced sympathetic tone, inflammation and blood pressure in the SHR (20). It is also well established that sympathetic hyperactivity occurs at the level of the peripheral ganglia themselves in the SHR, which exhibit greater stimulation-evoked noradrenaline release (27, 28), and show increased firing responsiveness to a given electrical stimulus (29). In neuropathic pain (30) and post-MI (31–33), inflammatory reactions induce states of local peripheral neuronal activity, mediated by cytokines and other neuronal growth factors. Interestingly, following sensory nerve injury, macrophage-released IL-6 contributes to the sprouting of sympathetic nerve fibers into the dorsal root ganglia (34), suggesting this classic inflammatory mediator, which is positively associated with blood pressure in humans (35), could produce sympathetic hyperactivity.

We therefore tested the hypothesis that a pathological immune system reaction within the sympathetic ganglia of pre-hypertensive animals drives local neuronal hyperactivity. Here we show that macrophages can enhance the intracellular calcium responsiveness of sympathetic neurons *in vitro*, which may underpin aspects of the increases in sympathetic hyperactivity seen in the early stages of hypertension.

## Methods and Materials

### Animals

Male Wistar and Spontaneously Hypertensive rats were purchased from Envigo, UK and housed in the local Biomedical Services Building prior to experimental use. Rats were used mainly at the age of 3–4 weeks, as this is an age at which the SHR exhibits dysautonomia, but this is not yet confounded by any increase in arterial blood pressure (36, 37). These SHRs are therefore termed “pre-hypertensive SHRs”.

To excise stellate ganglia for cell culture, rats were culled using an approved UK Home Office Schedule 1 method involving overdose of pentobarbitone (under isoflurane anesthesia), followed by exsanguination. In cases where a pure blood sample was required for co-culture with stellate neurons, a needle was inserted into the heart of terminally anesthetized rats, and ~1 ml of blood was withdrawn into a syringe containing ~100 μL EDTA solution (100 mM). Finally, to prepare tissues for flow cytometry, as tissue-resident leukocytes were to be examined, it was necessary to flush out blood leukocytes from the circulation. To achieve this, a small incision was made in the right atria of terminally anesthetized animals, and then 50–100 ml cold PBS containing 10 U/ml heparin was injected into the ventricles. Blood samples for flow cytometry were obtained by collecting some of the blood flushed out by this method.

### Cell Culture

Cleaned ganglia were cut into ~6 pieces each before enzymatic digestion in collagenase IV (1 mg/ml in L-15) for 25 min, followed by trypsin (2 mg/ml in Ca^2+^- and Mg^2+^-free Hank's Balanced Salt Solution; Merck or Thermofisher, both US) for 30 min; both at 37°C. Digested ganglia were then washed in blocking medium (Neurobasal Plus medium with 10% heat-inactivated fetal bovine serum and 1% penicillin/streptomycin; all Thermofisher, US; penicillin/streptomycin also Merck, US) at room temperature, twice for 5 min each. Next, the ganglia were mechanically triturated in complete neuronal medium (Neurobasal Plus medium with 2% B-27 supplement, 1.5 mM Glutamax, 5 ng/ml 2.5S NGF and 0.5% penicillin/streptomycin; all Thermofisher, US except NGF, Merck US) using two glass Pasteur pipettes in series, the second, fire-polished to narrow the opening. The resultant single-cell suspension was plated onto poly-D-lysine (0.1 mg/ml; Merck, US) and laminin- (0.048 mg/ml; Thermofisher, US) coated 6 mm glass coverslips in 4-well culture plates. These were incubated at 37°C under 5% CO_2_ for ~48 h prior to experimentation.

For some experiments ganglia were co-cultured with blood-derived leukocytes. These were prepared from blood samples taken from 3 to 4 week old Wistar rats and SHRs by transthoracic cardiac puncture, as detailed previously. Using this method 1–2 ml of fresh blood could be obtained per 3–4 week old animal. 1 ml of each sample was added to 10 ml of eBioscience red blood cell lysis buffer (Thermofisher, US) as per the manufacturer's instructions, and incubated at room temperature for 10 min on a shaker. To arrest the reaction, 10 ml of DMEM containing 10% FBS was added to each sample and the suspensions were centrifuged at 400 rcf for 4 min to obtain a pellet. Blood cell suspensions were then either depleted of monocytes using clodronate liposomes (Encapsula Nanosciences, US), according to a protocol developed by Claassen et al. (38), or incubated with PBS-containing liposomes as a control. The pellets were resuspended in the ganglionic culture medium containing either 100 μl clodrosomes, or the same concentration of the control liposomes, and incubated at 37°C for 1–2 h. After this the cells were pelleted and resuspended in fresh ganglionic culture medium and this suspension was used to culture the ganglionic explants. The products of each original 1 ml blood sample were resuspended in 2 ml of such medium; this being split across two ganglia so that each ganglion received approximately the number of leukocytes derived from 0.5 ml of rat blood. SHR blood may contain a higher concentration of leukocytes than that of Wistar rats (39–41), so this difference would therefore be incorporated in the culture conditions of ganglia treated with blood from each respective strain.

The following protocol was adapted from Muschter et al. (42). Tibiae and femurae were extracted from 3 to 4 week old Wistar rats, cleaned of soft tissue and stored in PBS at 4°C for up to 24 h. These were then sprayed with 70% ethanol and one at a time the ends of each bone were cut off with a scalpel. A syringe and 25 G needle were used to flush out the bone marrow into a petri dish, using ~6 ml cold PBS. The combined effluent from all bones was then passed through a 70 μm filter and collected in a 50 ml falcon tube, before being centrifuged at 300 rpm for 5 min. The cell pellet was resuspended in complete BMDM media (Dulbecco's Modified Eagle's Medium with 10% heat-inactivated fetal bovine serum, 1% penicillin/streptomycin and 20 ng/ml recombinant rat M-SCF), 4 ml/bone. The cells were plated in 10 cm petri dishes, 8 ml per dish, and incubated at 37°C/5% CO_2_. Four days later half the media was removed and replaced with fresh BMDM. Two days after this, all media was removed and the plates washed with PBS, before ~4 ml TrypLE (Thermofisher, US), or EDTA (2 mM in PBS; where cells were being processed for flow cytometry) was added to each. After 3–4 min once the cells had detached the suspension was removed and combined with an equal volume of α-MEM + 10% FBS media, and this was then centrifuged at 300 rpm for 5 min. Cells were then plated onto the required format, for example, 6 mm glass coverslips, re-suspended and prepared for flow cytometry.

Co-cultures of stellate neurons and blood leukocytes or BMDMs were prepared on 6 mm poly-D-lysine/laminin-coated coverslips. For leukocyte co-culture, stellate neurons were prepared in the same way as for their solitary culture, but in half the normal volume of medium. Blood leukocytes, prepared in the same way as described above for culture with whole ganglia, with either clodronate or PBS-filled liposomes, were then added to the neurons in an equal volume of neuronal culture medium. To separate these cells from the liposomes, after the incubation 10 ml of Neurobasal plus containing 21.5% Optiprep (Merck, US) was added to each ml of cell suspension, and the mixture centrifuged at 400 g for 15 min with no brake. The liposomes floated to the top while the cells formed a pellet at the bottom. These were resuspended in neuronal culture medium. Each ml of blood provided leukocytes for three wells of four 6 mm coverslips.

In the case of BMDM co-culture, the neurons were once again plated in half the normal volume of culture medium, and day 6 harvested BMDMs were added to this in the same volume of media, but containing 40 ng/ml M-CSF, to produce a final concentration of 20 ng/ml. These BMDMs had been previously cultured in 10 cm dishes at a density of 2 bones per dish, and were plated at an approximately equivalent density, adjusting for well surface area.

All co-culture preparations were incubated at 37°C, 5% CO_2_ for 48 h before imaging.

### Calcium Imaging

Stellate neurons were plated onto 6 mm laminin and poly-D-Lysine coated glass coverslips. Prior to imaging, these were incubated in culture medium containing 2 μM Fura-2 AM, at 37°C for 30 min, before being washed with Tyrode's solution (containing, in mM: NaCl 135, KCl 4.5, HEPES 20, Glucose 11, CaCl_2_ 2, MgCl_2_ 1) three times for 5 min each. The coverslips were then imaged in a 100 μL, gravity-fed perfusion chamber, in 37°C Tyrode's solution at a flow rate of ~3–4 ml/min. An inverted Nikon microscope, with a 40 × oil-immersion objective, was used to obtain the images, which were captured by QICLICK digital CCD camera, using Optofluor QICLICK software.

Neurons were identified by their large size and thick borders, as compared to other cells present on the coverslips, and only those which appeared healthy, showing no obvious signs of damage or blebbing, were selected for imaging. Images were captured every 2 s, during which the coverslips were excited sequentially at wavelengths of 355 and 380 nm, and the emission intensities at 510 nm recorded. Background 510 nm emissions were subtracted from that of the cells for both excitation wavelengths in each image, and the resultant values used to calculate the 355/380 ratio. This was then normalized to a baseline ratio for each cell, which was taken as the average of the final 5 images prior to addition of the first treatment.

### Flow Cytometry

All rat tissue samples were first prepared for subsequent staining and flow cytometric analysis or sorting. In the case of sympathetic ganglia, the cut ganglion pieces underwent simultaneous enzymatic digestion in HBSS with hyaluronidase (1,000 U/ml), collagenase II (1 mg/ml) and DNase I (5 U/ml) for 30 min at 37°C. Subsequently these were centrifuged at 400 rcf for 4 min, the supernatant was discarded, and then the tissue was mechanically triturated in 1 ml PBS using two glass Pasteur pipettes in series, the second fire-polished to narrow the opening.

Heparinised blood samples, obtained during cardiac perfusion, were passed through a 70 μm filter and centrifuged at 400 rcf for 4 min. Next these were resuspended in ~10 ml red blood cell lysis buffer and left at room temperature for 10–15 min, before the reaction was stopped by addition of an equal volume of FACS buffer, and the whole suspension was centrifuged again at the same settings as before. The pellet was then resuspended in PBS, spun down at 400 rcf for 4 min once more, before a final resuspension in fresh PBS. For experiments in which monocyte-macrophages were to be isolated by FACS, blood samples were instead obtained from rats by cardiac puncture under terminal anesthesia, with EDTA used as the anti-coagulant.

Renal tissue was prepared for flow cytometry using a protocol adapted from Rubio-Navarro et al. (43). Pairs of kidneys were stripped of their capsules by hand, the hila were removed. The remaining renal tissue was finely cut up using scissors and then pushed through a 40 μm filter using the plunger from a 10 ml syringe, the resultant pulp being washed through with 15 ml of FACS buffer. The filtered suspension was centrifuged at 400 rcf for 4 min and resuspended in ~15 ml red blood cell lysis buffer, which was left at room temperature for 90 s, before lysis was terminated by dilution with 15 ml of FACS buffer. The solution was centrifuged under the same settings as before, resuspended in 10 ml PBS, and 1 ml of this was taken forward for analysis.

The resultant single-cell suspensions were stained with 0.5 μL eBioscience™ Fixable *Via*bility Dye eFluor™ 780 (Thermofisher, US) for 30 min at 4°C, and then 1 ml of FACS buffer was added, and the suspension centrifuged at 400 rcf for 4 min to wash off the dye. The cells were next incubated for 10 min in anti-rat CD32 (1:100 in FACS buffer) at 4°C to prevent FcR-mediated antibody binding, prior to incubation for 30 min in appropriate panel of fluorescently-tagged flow cytometry antibodies.

For flow cytometry, a BD LSRFortessa™ X-20 was used, equipped with 5 lasers, and a total of 18 filter sets, each corresponding to an emission wavelength on a specific laser. Single color controls were prepared using compensation beads according to the manufacturer's instructions, and FMO controls were also used for each antibody panel to aid gating. Data were later processed using FlowJo 10 software.

### Immunohisto-/Cyto-Chemistry

Clean stellates were immersed in cold 4% PFA (Alfa Aesar, US) and stored at room temperature on a rotator for ~1–2 h; spleens were incubated in the same solution overnight. For cryoprotection, the tissues were transferred to 15% sucrose (Merck, US) in PBS solution for ~6–12 h, or until the tissue had sunk, and subsequently put into 30% sucrose for the effect. The tissue was then embedded in OCT and stored at −20°C as required. 20 μm sections were cut on a cryostat, transferred to microscope slides, and returned to −20°C for further storage. In the case of cell culture staining, neuron and macrophage co-cultures were plated directly onto 35 mm glass-bottomed dishes, before fixation for 15 min in 2% PFA.

For staining, microscope slides were thawed in room temperature PBS and then the tissue circled with a PAP pen. The sections or cell-containing dishes were then incubated in a PBS-based blocking and permeabilisation solution (PBS with 3% bovine serum albumin, 2% goat serum, 1% Triton X-100 and 0.1% NaN_3_), for 1 hr, before incubation in primary antibody solution overnight at 4°C in a humid box. The following day the stained slides/dishes were washed three times each for 5 min in PBS and then incubated in secondary antibody and DAPI for 1 h at room temperature. Finally, the samples were washed three times for 5 min in PBS once again, prior to mounting using ProLong™ Gold Antifade Mountant (Thermofisher, US). Imaging was carried out on a confocal microscope within a day of mounting, and the recorded images processed using ImageJ software.

### Statistical Analysis

All statistical analyses were performed using Graphpad Prism 8 software. All data analyzed in this publication were treated as continuous. Data normality was examined using Anderson-Darling, D'Agostino and Pearson, Shapiro-Wilk and Kolmogorov-Smirnov tests, and parametric or non-parametric tests selected based on the outcome of these. For comparison of two groups Welch's *t*-tests (parametric) or Mann-Whitney tests (non-parametric) were employed, with the two-step set up method of Benjamini, Krieger and Yekutieli used to produce *q*-values to account for multiple comparisons. Where three of more groups were to be compared, Brown-Forsythe and Welch's ANOVAs (parametric) or Kruskall-Wallis tests (non-parametric), with appropriate *post-hoc* tests for multiple comparisons were used. Results were considered statistically significant where *p* < 0.05.

## Results

### Pre-hypertensive SHR Neurons Exhibit Larger Ca^2+^ Transients in Response to Nicotinic Stimulation or High K^+^ Induced-Depolarization

**Figure 1:**
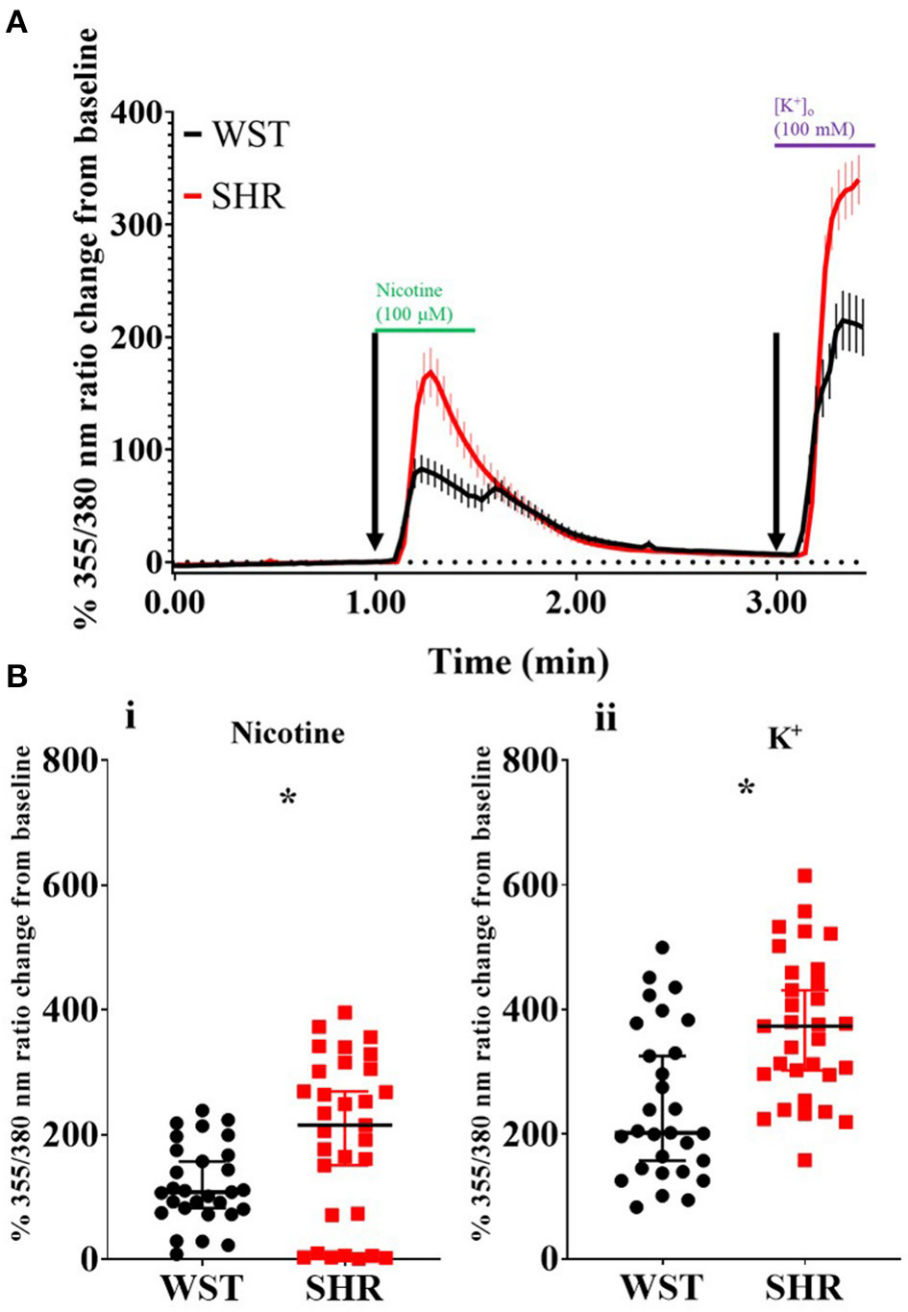
Wistar (*n* = 29 neurons, 5 cultures, 2–4 rats per culture) and SHR (*n* = 31 neurons, 6 cultures, 2–4 rats per culture) stellate neuron Ca^2+^ transients in response to pharmacological stimulation. **(A)** Mean trace (with SEM) of stellate neuron [Ca^2+^]_i_ during the experimental protocol involving exposure to 100 μM nicotine, followed by 100 mM K^+^. **(B)** Peak stellate neuron [Ca^2+^]_i_ transient in response to nicotine (i) and K^+^ (ii). Peak response data are presented as median with IQR; * signifies *p* < 0.05 for Wistar vs. SHR, Mann-Whitney test.

It has been previously reported that pre-hypertensive SHR stellate neurons show enhanced calcium transients ([Ca^2+^]_i_) compared to Wistar neurons, when depolarized using high [K^+^]_o_ Tyrode's solution (36). We first confirmed this while also examining the relative responses of these neurons to a more physiological stimulation with 100 μM nicotine. In both cases, the SHR neuron responsiveness was greater than that of Wistar neurons (expressed as % ratio change from baseline): nicotine: 194.5 ± 23.4 vs. 119.5 ± 11.87, *p* = 0.022; K^+^: 369.7 ± 20.74 vs. 246.1 ± 22.51, *p* = 0.00020 (Figure 1). Since the nicotinic response mimicked physiological post-ganglionic neuron synaptic activation, and also showed a difference between Wistar and SHR, the [Ca^2+^]_i_ response was taken forward as a surrogate measure of stellate neuronal activity.

### Whole Blood Leukocytes Increase Wistar, but Not SHR Responsiveness to Nicotinic Stimulation

**Figure 2:**
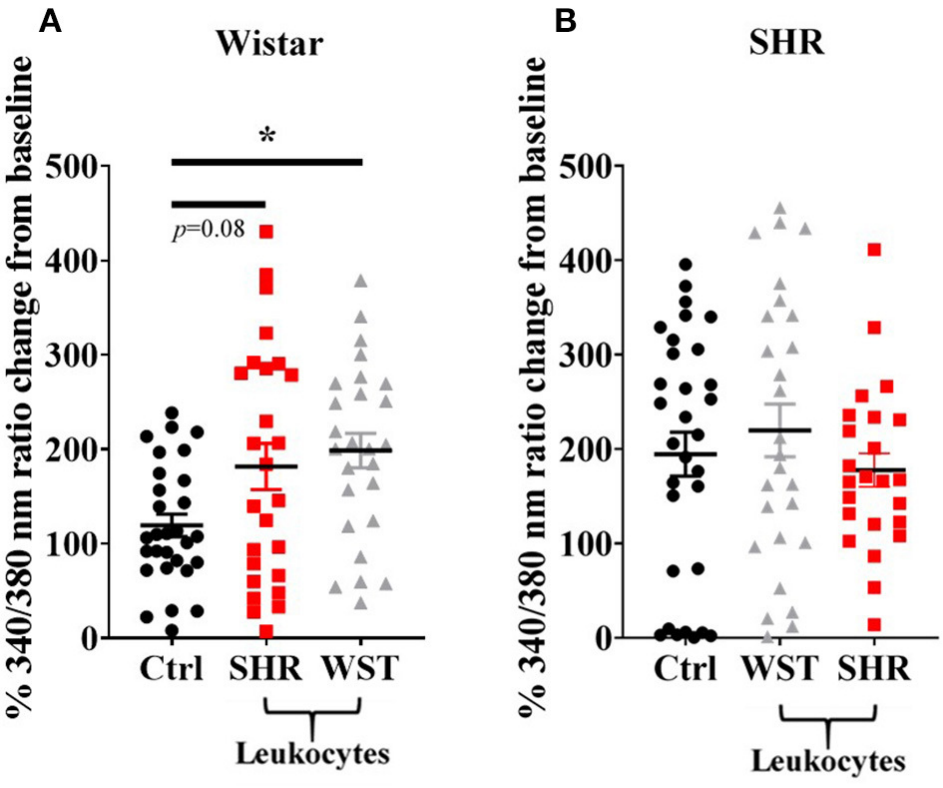
Peak [Ca^2+^]_i_ transient responses of stellate neurons co-cultured with blood leukocytes in response to 100 μM nicotine: **(A)** Wistar neurons (with SHR leukocytes: *n* = 26 neurons, 3 cultures, 2–3 rats per culture; with Wistar leukocytes: *n* = 26 neurons, 3 cultures, 2–3 rats per culture) and **(B)** SHR neurons (with Wistar leukocytes: *n* = 27 neurons, 3 cultures, 2–3 rats per culture; with SHR leukocytes: *n* = 24 neurons, 3 cultures, 2-3 rats per culture). Data are presented as mean ± SEM; * signifies *p* < 0.05 between the indicated groups; normal data were analyzed using Brown-Forsythe and Welch's ANOVAs with Dunnett's T3 multiple comparisons test, while non-normal data were analyses with a Kruskall-Wallis test followed by Dunn's multiple comparisons test.

Co-culturing Wistar stellate neurons with their own blood leukocytes significantly increased their responsiveness to nicotine. With SHR leukocytes there was a clear trend to this effect (Figure 2), although not significant (*p* = 0.080). However, the nicotinic response with SHR leukocytes did not differ significantly from that with the Wistar leukocytes, where these responses were much closer in magnitude than the baseline and SHR leukocyte co-culture. By contrast, SHR neurons showed no differences when co-cultured with these leukocytes (Figure 2).

### The SHR Exhibits a Shift in the Proportions of the Classical and Non-classical Monocyte-Macrophage Subsets

Given that leukocytes can potentiate stellate neuron responsiveness to nicotinic stimulation, we next examined the immune cell environment of the stellate ganglia in both strains of rat. Ganglia were separated into single cell suspensions, stained for markers of the main leukocyte populations, and analyzed using flow cytometry. As the ganglia were of different sizes, and those of the SHR tended to be smaller in size than Wistar ganglia, cell counts were normalized to the total number of single cells recorded for each sample, and expressed as the number per 1,000 single cells.

**Figure 3:**
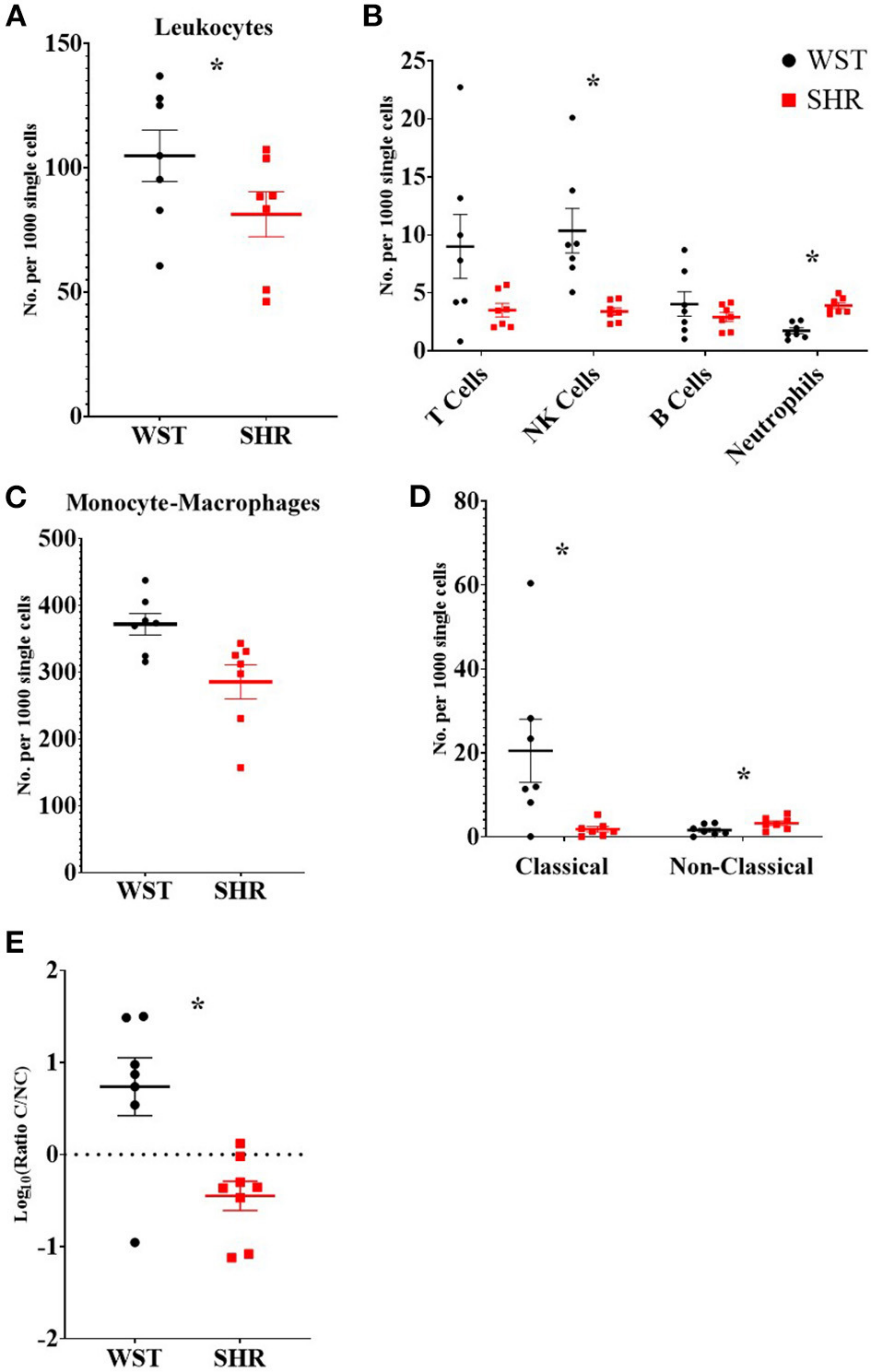
Numbers of different leukocyte populations found within the stellate ganglia of 3-week old Wistar rats (*n* = 7 samples, 2–3 rats per sample) and pre-SHRs (*n* = 7 samples, 2–3 rats per sample). **(A)** Numbers of leukocytes. **(B)** Stratification of CD45^+^ cells into major leukocyte populations; monocyte-macrophages shown in **(C)**. **(D)** Classical and non-classical monocytes-macrophages. **(E)** Ratio of classical/non-classical monocyte-macrophages. In **(A–D)** the values are expressed as numbers per 1,000 single cells to allow comparison between samples which contained a different total number of cells. In **(E)** the ratio is presented as log_10_ to allow visualization of the size of the ratio in either direction. Data are expressed as mean ± SEM. * signifies *p* < 0.05 WST vs. SHR; normal data were analyzed using Welch's t-tests and multiple comparisons accounted for using *q* values determined by the two-stage set-up method of Benjamini, Krieger and Yekutieli.

There were significantly more leukocytes (defined at CD45^+^) in the Wistar compared to SHR stellates (372 ± 16.1 vs. 285 ± 25.5 per 1,000 single cells; *p* = 0.014; *q* = 0.025; Figure 3A). Part of this increase in leukocytes was accounted for by increased numbers of NK cells (10.3 ± 1.91 vs. 3.39 ± 0.329 per 1,000 single cells; *p* = 0.0037; *q* = 0.0097; Figure 3B) in the Wistar strain, while the SHR had higher numbers of neutrophils (3.90 ± 0.259 vs. 1.73 ± 0.253; *p* < 0.0001; *q* = 0.00033; Figure 3B).

**Figure 4:**
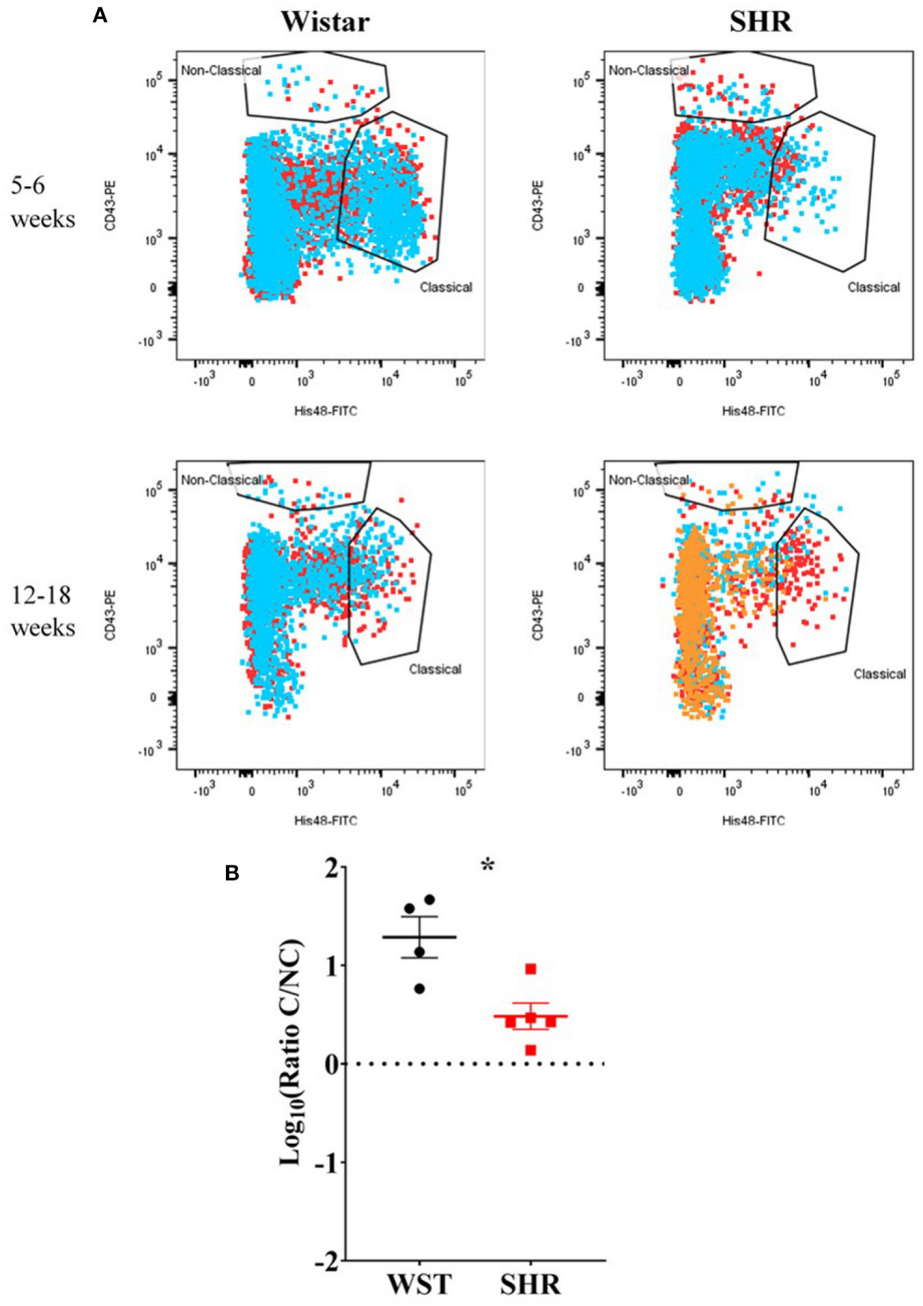
Monocyte-macrophages from the stellate ganglia of SHR *n* = 5 samples, 3 rats per sample, and age-matched Wistars (*n* = 4 samples, 3 rats per sample) at 5–6 weeks (developing hypertension) and 12–18 weeks (established hypertension). **(A)** Flow cytometry plots of all samples; each color represents a separate sample of ganglia from 2–3 rats. **(B)** Classical/non-classical monocyte-macrophage ratio from all 5-18 week old animals; Data are expressed as mean ± SEM. *signifies *p* < 0.05 WST vs. SHR, Welch's t-test.

Despite no differences in the overall numbers of monocyte-macrophages between the two strains (Figure 3C), there was a striking difference in the proportions of the subtypes between these two strains: the ratio of classical (His48^high^/CD43^low^) to non-classical (CD43^high^/His48^low−int^) monocyte-macrophages was significantly higher in the Wistar (13.8 ± 4.77 vs. 0.551 ± 0.148; *p* = 0.0082; Figure 3E). This appeared to made up of both more classical (20.52 ± 7.54 vs. 1.81 ± 0.662; *p* = 0.029; *q* = 0.039; Figure 3D), and fewer non-classical (1.60 ± 0.476 vs. 3.24 ± 0.556; *p* = 0.047; *q* = 0.0497; Figure 3D), monocyte-macrophages in the Wistar compared to SHR. This finding was also replicated in the stellate ganglia of older (5–6 week and 12–18 week old) animals (Figure 4).

The same flow cytometry panel was then employed on two other SHR sympathetic ganglia to examine whether this was an SNS-wide phenomenon: the coeliac ganglion (which innervates the kidney, so is likely important to hypertensive pathophysiology) and the superior cervical ganglion (innervating the head, so likely has minimal impact on hypertension, except possibly *via* bone-marrow effects (44).

**Figure 5:**
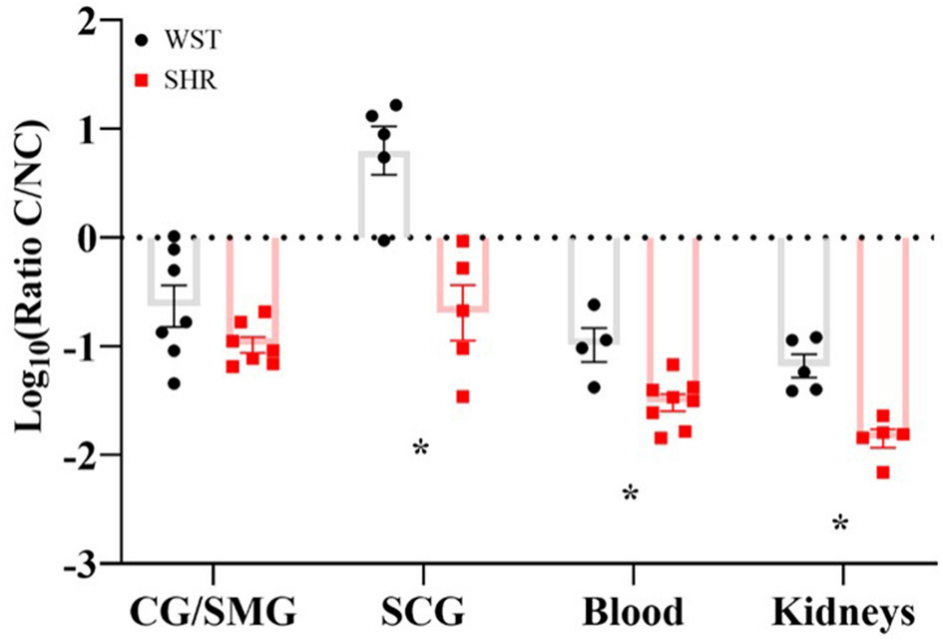
Classical/non-classical monocyte-macrophage ratios in the coeliac/superior mesenteric ganglia (CG/SMG; Wistar: *n* = 7 samples, 2 rats per sample; SHR: *n* = 7 samples, 2-4 rats per sample), superior cervical ganglia (SCG; in both strains: *n* = 5 samples, 2 rats per sample), blood (Wistar *n* = 4 samples, SHR *n* = 8 samples, 1 rat per sample) and kidneys (both strains *n* = 5 samples, 1 rat per sample) of 3-week old Wistar rats and pre-SHRs. Data are expressed as mean ± SEM. * signifies *p* < 0.05 WST vs. SHR, Welch's t-tests with *q* values determined by the two-stage set-up method of Benjamini, Krieger and Yekutieli.

In the SCG, the Wistar ganglia had a significantly higher classical/non-classic monocyte-macrophage ratio (8.99 ± 2.46 vs. 0.359 ± 0.148; *p* = 0.0023; *q* = 0.0012), while in the coeliac ganglion there was a clear trend in the same direction (0.391 ± 0.145 vs. 0.112 ± 0.021), although not significant (*p* = 0.12; *q* = 0.032; Figure 5). A similar monocyte-macrophage subset ratio result was apparent in the whole blood compared between strains (Wistar: 0.123 ± 0.0422 vs. SHR: 0.0337 ± 0.00600; *p* = 0.0323, *q* = 0.011; Figure 5), suggesting this phenomenon is not specific to the SNS, but occurs systemically. Finally, this was also the case in the kidneys (Wistar: 0.0742 ± 0.107 vs. SHR: 0.0151 ± 0.0853; *p* = 0.0014; *p* = 0.0012; Figure 5), typically referred to as the “final common node of hypertensive pathophysiology” (Crowley and Coffman, 2014). However, no differences in any of the other leukocyte populations were detected in either the blood or kidneys. Bone marrow-derived macrophages potentiate stellate neuron responsiveness to nicotinic stimulation.

**Figure 6:**
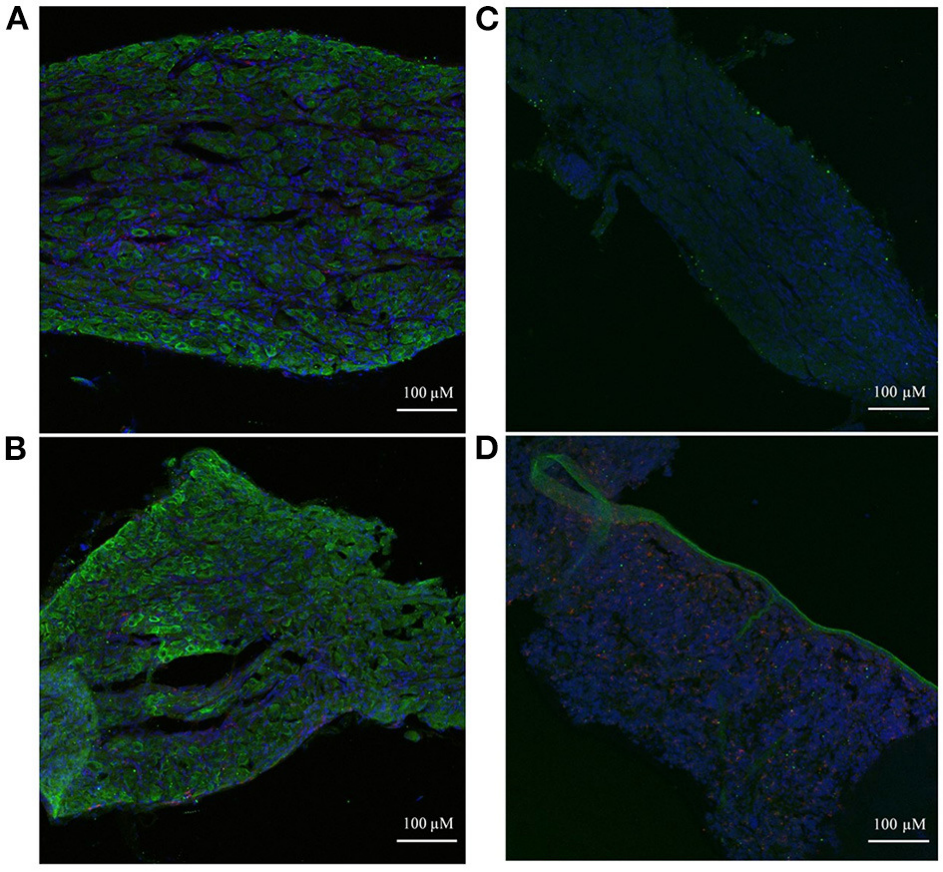
Immunohistochemical images of sympathetic neurons (TH) and macrophages (CD68) in 3-week old Wistar and pre-SHR rat tissue. **(A)** Wistar stellate ganglion, **(B)** SHR stellate ganglion. These ganglia comprise a large number of sympathetic neurons (green) with some small macrophages (red) amongst them. The acellular spaces within the tissue likely reflect empty blood vessels following cardiac perfusion. **(C)** Wistar stellate ganglion stained with only DAPI and the secondary antibodies, serving as a negative control. There appears to be some 488 nm auto-fluorescence, but the tissue clearly lacks the cellular pattern of **(A)**. **(D)** Wistar spleen stained as a positive control for CD68^+^ macrophages; many punctate cells appear similarly to those of **(A,B)**. A large TH^+^ ribbon which penetrates the rest of the tissue is interestingly also present, likely representing a sympathetic nerve fiber.

Immunohistochemistry was used to examine the spatial localization of the stellate ganglion monocyte-macrophages relative to the neurons. This revealed large numbers of spindle-shaped CD68^+^ cells, scattered amongst the TH^+^ sympathetic neurons, of both the Wistar (Figure 6A) and SHR (Figure 6B) stellates. Additionally, the appearance of large nucleus-free cavities within the tissue suggested that the ganglionic vasculature had been substantially flushed of circulating cells. A no primary antibody negative control (Figure 6C), and a splenic sample serving as a positive control for CD68^+^ macrophages (Figure 6D) are also shown.

### Co-culture With Bone Marrow-Derived Macrophages Enhances Wistar Stellate Neuron Nicotinic Responsiveness, but SHR Neurons Are Only Further Enhanced by Co-culture With Their Own Strain of Macrophages

As the most striking difference between the pre-hypertensive SHR and Wistar rats was the altered monocyte-macrophage subset ratio, we examined the effect of co-culturing stellate neurons from these strains with macrophages (bone-marrow derived) from either strain. We had previously attempted to FAC-sort macrophages from SHR and WST stellate ganglia, but unfortunately as there are such low numbers of these cells it is impossible to extract enough for culture. For this reason, we used bone marrow-derived macrophages (BMDMs), which arise from the same bone marrow monocyte precursor thought to give rise to the stellate monocyte-macrophages (45).

**Figure 7:**
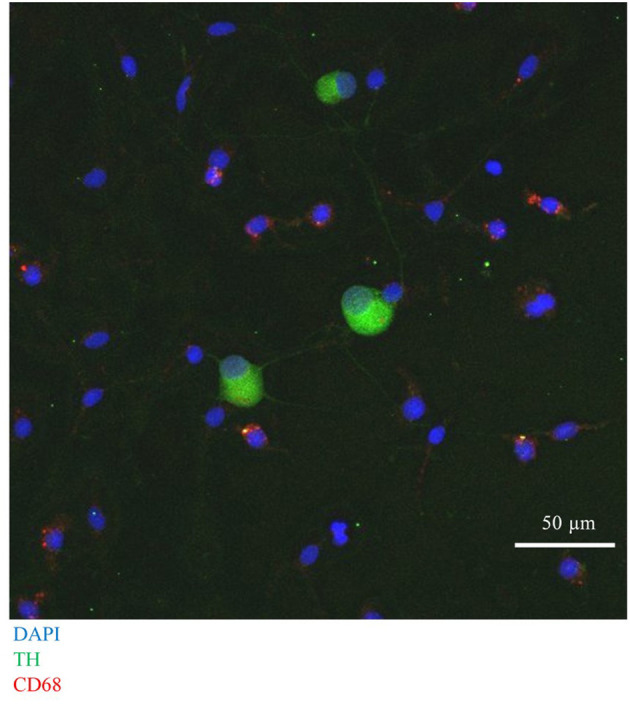
Immunocytochemical image of Wistar stellate neurons (TH^+^, green), with BMDMs (CD68^+^, red).

First, we confirmed successful co-culture of sympathetic stellate neurons (TH^+^) with BMDMs (CD68^+^), using immunocytochemistry, which revealed the presence of both cell types in close proximity (Figure 7).

**Figure 8:**
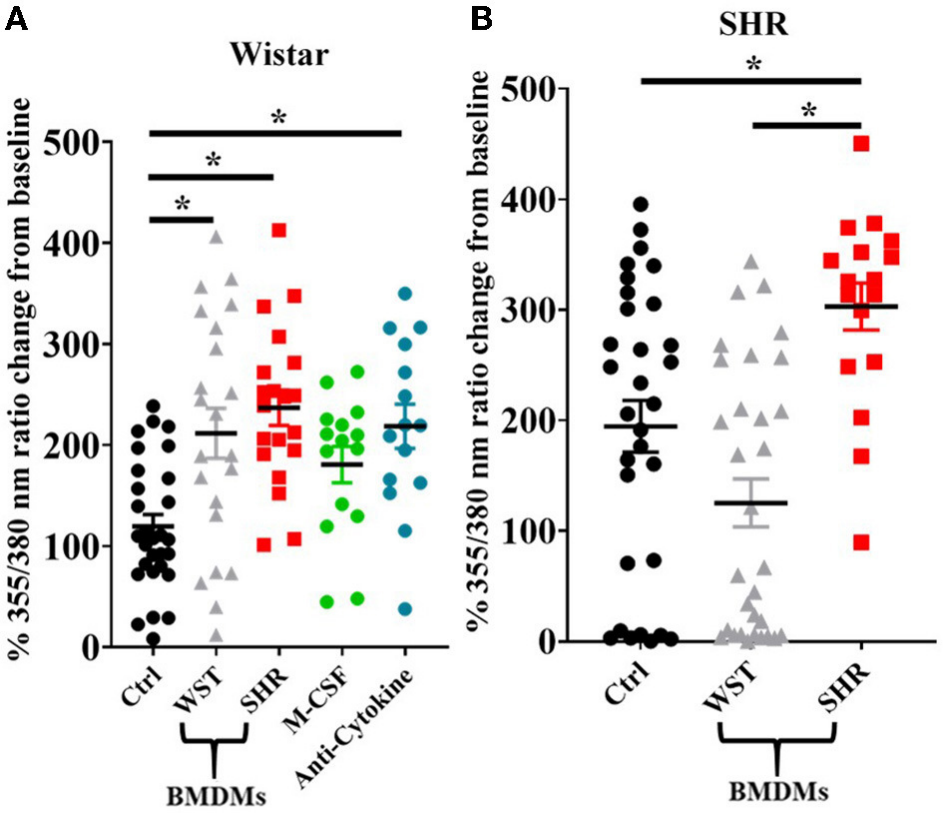
Peak [Ca^2+^]_i_ transient responses of stellate neurons co-cultured with BMDMs in response to 100 μM nicotine: **(A)** Wistar neurons (with Wistar BMDMs: *n* = 20 neurons, 2 cultures, 2 rats; with SHR BMDMs: *n* = 22 neurons, 1 culture, 2 rats; M-CSF control: *n* = 15 neurons, 2 cultures, 2–4 rats per culture, anti-cytokine: *n* = 15, 2 cultures, 2 rats per culture) and **(B)** SHR neurons (with Wistar BMDMs: *n* = 31 neurons, 3 cultures; with SHR BMDMs: *n* = 17 neurons, 2 cultures). Data are presented as mean ± SEM; * signifies *p* < 0.05 between the indicated groups; normal data were analyzed using Brown-Forsythe and Welch's ANOVAs with Dunnett's T3 multiple comparisons test, while non-normal data were analyses with a Kruskall-Wallis test followed by Dunn's multiple comparisons test.

Upon BMDM co-culture, as with the blood leukocytes, the Wistar stellate neurons showed increased nicotine responsiveness in the presence of BMDMs from either strain, while the SHR neurons only showed an increased response when cultured with their own BMDMs, not those of Wistar rats (Figures 8A,B). As the BMDM co-culture required addition of M-CSF to keep the BMDMs alive, control experiments were performed in which Wistar neurons alone were cultured with the same concentration of this factor. These neurons did not display a nicotinic response differing significantly from baseline, and there was no significant difference between this response and those from the BMDM co-cultures (Figure 8A).

As the presence of BMDMs increased Wistar stellate neuron responsiveness to nicotine, an attempt was made to identify potential mediating factors. Macrophages contribute to neuronal hyperactivity through release of pro-inflammatory cytokines, therefore we tested the effect of combined blockade of the classical triad of inflammatory cytokines: TNF-α, IL-1β and IL-6. However, the nicotinic responsiveness of Wistar stellate neurons co-cultured with Wistar BMDMs was still enhanced in the presence of antibodies targeted against TNF-α (100 ng/ml), IL-1β (140 ng/ml) and IL-6 (833 ng/ml) (Figure 8A).

The results of all such co-culture experiments are summarized in Table 1.

**Table 1:** Summary of stellate neuronal [Ca^2+^]_i_ transient responsiveness to nicotinic stimulation in co-culture experiments.

		**Control**	**Leukocytes**		**BMDMs**		
			**WST**	**SHR**	**WST**	**SHR**	**WST with TNF-α, IL-1β and IL-6 blockade**
Stellate neuron strain	WST	Baseline	+	Baseline^a^	+	+	+
	SHR	+	+	+	+	++	Not tested

*Wistar neurons in monoculture are taken as the baseline and all other permutations described relative to this.*

a *The nicotinic responsiveness of Wistar stellate neurons co-cultured with SHR leukocytes did not quite statistically significantly differ from baseline (p = 0.080). For reasons discussed in Chapter V, it seems possible that this result was a type II error and that SHR leukocytes should increase Wistar neuron [Ca^2+^]_i_ transient responsiveness to stimulation. This is due to the observations that: (1) the nicotinic response of Wistar stellate neurons co-cultured with SHR leukocytes did not differ significantly from that of those cultured with Wistar leukocytes, which were themselves significantly different from baseline; (2) the responses of these two populations were much closer together in magnitude than that of the neurons cultured with SHR leukocytes compared to baseline; and (3) SHR leukocytes significantly increased the responsiveness of Wistar neurons to high [K^+^]_o_ depolarization*.

## Discussion

This study reports three novel findings: (1) The SHR sympathetic ganglia, blood and kidneys display a reduced classical/non-classical monocyte ratio, when compared to sympathetic ganglia from age matched Wistar rats. (2) Co-culturing Wistar blood leukocytes with Wistar stellate neurons increases their [Ca^2+^]_i_ transient to nicotinic stimulation, making them phenocopy those of the SHR, although SHR leukocytes did not significantly affect pre-hypertensive SHR neurons. (3) Co-culturing either Wistar or SHR bone marrow-derived macrophages (BMDMs) with Wistar stellate neurons increases their [Ca^2+^]_i_ transient to nicotinic stimulation, but only SHR (and not Wistar) BMDMs further enhances the responsiveness of SHR neurons.

### Monocyte-Macrophage Subset Shift: A Feature of Hypertension?

The pre-SHR displays a lack of classical monocyte-macrophages in its sympathetic ganglia, and a relative enrichment of non-classical monocyte-macrophages. This lower classical/non-classical monocyte ratio is observed in the blood and kidneys of the pre-SHR. This was the most striking and consistently observed difference between the SHR and Wistar strains.

What is the significance of this finding? Many other inflammatory-related diseases feature a relative enrichment of non-classical blood monocytes, including: systemic lupus erythomatosis (46, 47), sepsis (46), obesity and metabolic syndrome (48), and rheumatoid arthritis (49). Furthermore, these cells seem to exhibit a pro-inflammatory phenotype (46, 50–52), which has been implicated in these disease states, suggesting the altered monocyte-macrophage ratio might also be an immunological feature of essential hypertension.

### Role of Macrophages in Sympathetic Neuron Hyperactivity?

Non-classical monocytes are major producers of inflammatory cytokines (46, 50, 52) and possess a strong ability to activate naïve T cells (51). In each of these cases the non-classical monocytes were observed to be the most potent monocyte subset. Zhu et al. (47) also showed CD16^+^ monocytes were strong activators of B cells, although they grouped non-classical and intermediate monocytes together, so a specific non-classical effect cannot be determined. Furthermore, looking at more specific disease contexts, CD16^+^ from SLE patients display enhanced inflammatory attributes, and in the murine serum-transfer rheumatoid arthritis model, clodronate-depletion of monocytes and replacement with non-classical monocytes enhances disease, while replacement with classical ones delays it (53). By contrast, pro-phagocytic, homeostatic roles for classical monocytes have been observed (46, 54).

If the SHR stellate has lost a homeostatic cell type, and gained a pathological one, this may interfere with local neuronal functioning. Local inflammation promotes states of local neuronal hyper-excitability in a number of contexts, many involving the sympathetic nervous system (20, 26, 30–33). Moreover, anti-TNFα treatment infliximab causes an increase in circulating non-classical monocytes in Crohn's disease patients (55), along with an increase in all CD16^+^ monocytes in rheumatoid arthritis patients (56). The latter study showed a concomitant reduction in circulating CCL2 causing them to hypothesize that this increase in blood non-classical monocytes was due to reduced recruitment to inflamed tissues. However, CCL2 is mainly a chemoattractant for *classical* monocytes (57). The mechanism would thus have to involve reduced classical monocyte recruitment to the tissues and therefore reduced subsequent differentiation into non-classical monocytes. Since blood classical monocyte numbers increased in our study though, this seems unlikely.

In the pre-hypertensive SHR stellate ganglia of this study, there were higher relative numbers of neutrophils, an important inflammatory cell type, which are known to drive monocyte recruitment and polarization toward an inflammatory phenotype (58). Moreover, SHR neutrophils tend to produce higher levels of ROS (40, 59, 60), which tend to enhance inflammation. This certainly suggests some form of immunological reaction occurring in this tissue.

Very interestingly though, whole blood leukocytes in co-culture increase Wistar stellate neuron [Ca^2+^]_i_ transients, but not those from the SHR. Moreover, BMDMs of either strain increase Wistar neuron responsiveness to stimulation, while only SHR BMDMs increase that of SHR neurons. It seems unlikely (though not impossible) that these macrophages or other leukocytes interact physically with the neurons, at least to any substantial extent. We think it more probable that these immune cells release a factor (or group of factors), which sensitizes the sympathetic neurons. However, our multi-cytokine blockade experiment does not support a role for TNF-α, IL-1β or IL-6. The observation that SHR neurons are unaffected by whole blood leukocytes, may imply they have already been exposed to a sensitizing stimulus. Nevertheless, SHR BMDMs can still increase the activity of SHR stellate neurons (while those from Wistar rats cannot), suggesting a property specific to these SHR macrophages can promote the excitability of already hyperactive neurons, where their general leukocytes cannot. Taken together with the altered monocyte-macrophage composition of the SHR stellate ganglion, it is likely that macrophages play a role in SHR peripheral sympathetic hyperactivity.

## Data Availability Statement

The raw data supporting the conclusions of this article will be made available by the authors, without undue reservation.

## Ethics Statement

Ethical review and approval was not required for the animal study because the keeping and use of these animals was covered by the UK Home Office Project License (PPL) of D. J. Paterson: 30/3031 and P707EB251. The use of animals in the experiments of this publication complied with the University of Oxford Local Ethical Guidelines and the Animals (Scientific Procedures) Act 1986 of the United Kingdom.

## Author Contributions

OCN designed and performed the experiments, analyzed the data, and drafted the manuscript. DJP and AID helped design the experiments and edited the manuscript. All authors contributed to the article and approved the submitted version.

## Funding

This study was funded by the British Heart Foundation Centre of Research Excellence and a BHF programme grant, UK (*RG*/17/14/33085) awarded to DJP. OCN was funded by a BHF Ph.D. studentship. AID is supported by the European Research Council (ERC-2017-COG-771431), and by Wellcome (208576/Z/17/Z).

## Conflict of Interest

The authors declare that the research was conducted in the absence of any commercial or financial relationships that could be construed as a potential conflict of interest.

## Publisher's Note

All claims expressed in this article are solely those of the authors and do not necessarily represent those of their affiliated organizations, or those of the publisher, the editors and the reviewers. Any product that may be evaluated in this article, or claim that may be made by its manufacturer, is not guaranteed or endorsed by the publisher.

## References

[1] Grassi G. Assessment of sympathetic cardiovascular drive in human hypertension achievements and perspectives. Hypertension. (2009) 54:690–7. 10.1161/HYPERTENSIONAHA.108.119883 19720958

[2] Mancia G Grassi G. The autonomic nervous system and hypertension. Circ Res. (2014) 114:1804–14. 10.1161/CIRCRESAHA.114.302524 24855203

[3] Grassi G Mark A Esler M. The sympathetic nervous system alterations in human hypertension. Circ Res. (2015) 116:976–90. 10.1161/CIRCRESAHA.116.303604 25767284 PMC4367954

[4] Grassi G Seravalle G Quarti-Trevano F. The ‘neuroadrenergic hypothesis' in hypertension: current evidence. Exp Physiol. (2010) 95:581–6. 10.1113/expphysiol.2009.047381 20008032

[5] Herring N Kalla M Paterson DJ. The autonomic nervous system and cardiac arrhythmias: current concepts and emerging therapies. Nat Rev Cardiol. (2019) 16:707–26. 10.1038/s41569-019-0221-2 31197232

[6] Grassi G Cattaneo BM Seravalle G Lanfranchi A. Baroreflex control of sympathetic nerve activity in essential and secondary hypertension. Hypertension. (1998) 31:68–72. 10.1161/01.HYP.31.1.68 9449393

[7] Grassi G Seravalle G Bertinieri G Turri C Dell'oro R Stella ML. Sympathetic and reflex alterations in systo-diastolic and systolic hypertension of the elderly. J Hypertens. (2000) 18:587–93. 10.1097/00004872-200018050-00012 10826562

[8] Smith PA Graham LN Mackintosh AF Stoker JB. Relationship between central sympathetic activity and stages of human hypertension. Am J Hypertens. (2004) 17:217–22. 10.1016/j.amjhyper.2003.10.010 15001194

[9] Floras JS Hara K. Sympathoneural and hemodynamic characteristics of young subjects with mild essential-hypertension. J Hypertens. (1993) 11:647–55. 10.1097/00004872-199306000-00009 8397244

[10] Yamada Y Miyajima E Tochikubo O Matsukawa T Shionoiri H Ishii M. Impaired baroreflex changes in muscle sympathetic-nerve activity in adolescents who have a family history of essential-hypertension. J Hypertens. (1988) 6:S525–8. 10.1097/00004872-198812040-00165 3241250

[11] Masuo K Mikami H Ogihara T. Sympathetic nerve hyperactivity precedes hyperinsulinemia and blood pressure elevation in a young, nonobese Japanese population. Am J Hypertens. (1997) 10:77–83. 10.1016/S0895-7061(96)00303-2 9008251

[12] Ferrier C Cox H Esler M. Elevated total-body noradrenaline spillover in normotensive members of hypertensive families. Clin Sci. (1993) 84:225–30. 10.1042/cs0840225 8382587

[13] Burns J Sivananthan MU Ball SG Mackintosh AF Mary DASG. Relationship between central sympathetic drive and magnetic resonance imaging-determined left ventricular mass in essential hypertension. Circulation. (2007) 115:1999–2005. 10.1161/CIRCULATIONAHA.106.668863 17389264 PMC3925820

[14] Levick SP Murray DB Janicki JS. Sympathetic nervous system modulation of inflammation and remodeling in the hypertensive heart. Hypertension. (2010) 55:270–U129. 10.1161/HYPERTENSIONAHA.109.142042 20048196 PMC2823485

[15] Lown B Verrier RL. Neural activity and ventricular-fibrillation. N Engl J Med. (1976) 294:1165–70. 10.1056/NEJM197605202942107 57572

[16] Fisher JP Young CN Fadel PJ. Central sympathetic overactivity: Maladies and mechanisms. Auton Neurosci Basic Clin. (2009) 148:5–15. 10.1016/j.autneu.2009.02.003 19268634 PMC2679852

[17] Julius S Valentini M. Consequences of the increased autonomic nervous drive in hypertension, heart failure and diabetes. Blood Press Suppl. (1998) 3:5–13. 10.1080/080370598438410-1 10321448

[18] Singh MV Chapleau MW Harwani SC. The immune system and hypertension. Immunol Res. (2014) 59:243–53. 10.1007/s12026-014-8548-6 24847766 PMC4313884

[19] Rodriguez-Iturbe B Pons H Johnson RJ. Role of the immune system in hypertension. Physiol Rev. (2017) 97:1127–64. 10.1152/physrev.00031.2016 28566539 PMC6151499

[20] Santisteban MM Ahmari N Carvajal JM Zingler MB Qi YF Kim S. Involvement of bone marrow cells and neuroinflammation in hypertension. Circ Res. (2015) 117:178–91. 10.1161/CIRCRESAHA.117.305853 25963715 PMC4490954

[21] Sesso HD Buring JE Rifai N Blake GJ Gaziano JM. C-reactive protein and the risk of developing hypertension. JAMA. (2003) 290:2945–51. 10.1001/jama.290.22.2945 14665655

[22] King DE Egan BM Mainous AG. Elevation of C-reactive protein in people with prehypertension. J Clin Hypertens. (2004) 6:562–8. 10.1111/j.1524-6175.2004.03577.x 15470285 PMC8109659

[23] Lakoski SG Cushman M Siscovick DS Blumenthal RS Palmas W Burke G. The relationship between inflammation, obesity and risk for hypertension in the Multi-Ethnic Study of Atherosclerosis (MESA). J Hum Hypertens. (2011) 25:73–9. 10.1038/jhh.2010.91 20944659 PMC4066617

[24] Jayedi A Rahimi K Bautista LE Nazarzadeh M Zargar MS. Inflammation markers and risk of developing hypertension: a meta-analysis of cohort studies. Heart. (2019) 105:686–92. 10.1136/heartjnl-2018-314216 30700522 PMC6588169

[25] Larabee CM Neely OC Domingos AI. Obesity: a neuroimmunometabolic perspective. Nat Rev Endocrinol. (2020) 16:30–43. 10.1038/s41574-019-0283-6 31776456

[26] Wu KLH Chan SHH Chan JYH. Neuroinflammation and oxidative stress in rostral ventrolateral medulla contribute to neurogenic hypertension induced by systemic inflammation. J Neuroinflammation. (2012) 9. 10.1186/1742-2094-9-212 22958438 PMC3462714

[27] Shanks J Manou-Stathopoulou S Lu CJ Li D Paterson DJ. Cardiac sympathetic dysfunction in the prehypertensive spontaneously hypertensive rat. Am J Physiol Heart Circ Physiol. (2013) 305:H980–6. 10.1152/ajpheart.00255.2013 23913706 PMC3798753

[28] Bardsley EN Davis H Buckler KJ. Neurotransmitter switching coupled to beta-adrenergic signaling in sympathetic neurons in prehypertensive states. Hypertension. (2018) 71:1226–38. 10.1161/HYPERTENSIONAHA.118.10844 29686017 PMC5959210

[29] Davis H Herring N Paterson DJ. Downregulation of M current is coupled to membrane excitability in sympathetic neurons before the onset of hypertension. Hypertension. (2020) 76:1915–23. 10.1161/HYPERTENSIONAHA.120.15922 33040619 PMC8360673

[30] Scholz J Woolf CJ. The neuropathic pain triad: neurons, immune cells and glia. Nat Neurosci. (2007) 10:1361–8. 10.1038/nn1992 17965656

[31] Zhou SM Chen LS Miyauchi Y Miyauchi M Kar S Kangavari S. Mechanisms of cardiac nerve sprouting after myocardial infarction in dogs. Circ Res. (2004) 95:76–83. 10.1161/01.RES.0000133678.22968.e3 15166093

[32] Hasan W Jama A Donohue T Wernli G Onyszchuk G Al-Hafez B. Sympathetic hyperinnervation and inflammatory cell NGF synthesis following myocardial infarction in rats. Brain Res. (2006) 1124:142–54. 10.1016/j.brainres.2006.09.054 17084822 PMC1769447

[33] Wernli G Hasan W Bhattacherjee A Van Rooijen N. Macrophage depletion suppresses sympathetic hyperinnervation following myocardial infarction. Basic Res Cardiol. (2009) 104:681–93. 10.1007/s00395-009-0033-3 19437062 PMC3692275

[34] Ramer MS Murphy PG Richardson PM. Spinal nerve lesion-induced mechanoallodynia and adrenergic sprouting in sensory ganglia are attenuated in interleukin-6 knockout mice. Pain. (1998) 78:115–21. 10.1016/S0304-3959(98)00121-3 9839821

[35] Fernandez-Real JM Vayreda M Richart C Gutierrez C Broch M Vendrell J. Circulating interleukin 6 levels, blood pressure, and insulin sensitivity in apparently healthy men and women. J Clin Endocrinol Metab. (2001) 86:1154–9. 10.1210/jcem.86.3.7305 11238501

[36] Li D Lee CW Buckler K Parekh A Herring N. Abnormal intracellular calcium homeostasis in sympathetic neurons from young prehypertensive rats. Hypertension. (2012) 59:642–U282. 10.1161/HYPERTENSIONAHA.111.186460 22252398 PMC3299568

[37] Dickhout JG Lee R. Blood pressure and heart rate development in young spontaneously hypertensive rats. Am J Physiol Heart Circ Physiol. (1998) 274:H794–800. 10.1152/ajpheart.1998.274.3.H794 9530190

[38] Claassen I Vanrooijen N Claassen E. A new method for removal of mononuclear phagocytes from heterogeneous cell-populations invitro, using the liposome-mediated macrophage suicide techniquE. J Immunol Methods. (1990) 134:153–61. 10.1016/0022-1759(90)90376-7 2147710

[39] Burba-Anczewska I. Leukocyte system in spontaneously hypertensive rats. Acta Physiol Pol. (1978) 29:353–8. 742369

[40] Schmidschonbein GW Seiffge D Delano FA Shen K. Leukocyte counts and activation in spontaneously hypertensive and normotensive rats. Hypertension. (1991) 17:323–30. 10.1161/01.HYP.17.3.323 1999363

[41] Reed JP Hendley ED. Blood-cell changes in spontaneously hypertensive rats are not all associated with the hypertensive phenotype. J Hypertens. (1994) 12:391–9. 10.1097/00004872-199404000-00009 8064163

[42] Muschter D Gottl C Vogel M Grifka J Straub RH. Reactivity of rat bone marrow-derived macrophages to neurotransmitter stimulation in the context of collagen II-induced arthritis. Arthritis Res Ther. (2015) 17:169. 10.1186/s13075-015-0684-4 26104678 PMC4496866

[43] Rubio-Navarro A Guerrero-Hue M Martn-Fernandez B Cortegano I Olivares-Alvaro E Heras NDL. Phenotypic characterization of macrophages from rat kidney by flow cytometry. J Vis Exp. (2016) 116:54599. 10.3791/54599 27805599 PMC5092211

[44] Scheiermann C Kunisaki Y Lucas D Chow A Jang JE Zhang D. Adrenergic nerves govern circadian leukocyte recruitment to tissues. Immunity. (2012) 37:290–301. 22863835 10.1016/j.immuni.2012.05.021 PMC3428436

[45] Pirzgalska RM Seixas E Seidman JS Link VM Sanchez NM Mahu I. Sympathetic neuron-associated macrophages contribute to obesity by importing and metabolizing norepinephrine. Nat Med. (2017) 23:1309–18. 29035364 10.1038/nm.4422 PMC7104364

[46] Mukherjee R Barman PK Thatoi PK Tripathy R Das BK. Non-Classical monocytes display inflammatory features: validation in Sepsis and Systemic Lupus Erythematous. Sci Rep. (2015) 5:13886. 10.1038/srep13886 26358827 PMC4566081

[47] Zhu HQ Hu FL Sun XL Zhang XY Zhu L Liu X. CD 16(+) monocyte subset was enriched and functionally exacerbated in driving T-cell activation and B-cell response in systemic lupus erythematosus. Front Immunol. (2016) 7:512. 10.3389/fimmu.2016.00512 27917174 PMC5116853

[48] Poitou C Dalmas E Renovato M Benhamo V Hajduch F Abdennour M. CD14. (dim)CD16(+) and CD14(+)CD16(+) monocytes in obesity and during weight loss relationships with fat mass and subclinical atherosclerosis. Arteriosclerosis Thromb Vasc Biol. (2011) 31:2322-U372. 10.1161/ATVBAHA.111.230979 21799175

[49] Lacerte P Brunet A Egarnes B Duchene B Brown JP. Overexpression of TLR2 and TLR9 on monocyte subsets of active rheumatoid arthritis patients contributes to enhance responsiveness to TLR agonists. Arthritis Res Ther. (2016) 18:10. 10.1186/s13075-015-0901-1 26759164 PMC4718023

[50] Wong KL Tai JJY Wong WC Han H Sem X Yeap WH. Gene expression profiling reveals the defining features of the classical, intermediate, and nonclassical human monocyte subsets. Blood. (2011) 118:E15–30. 10.1182/blood-2010-12-326355 21653326

[51] Liu BY Dhanda A Hirani S Williams EL Sen HN Estrada FM. CD14. (++)CD16(+) monocytes are enriched by glucocorticoid treatment and are functionally attenuated in driving effector T cell responses. J Immunol. (2015) 194:5150–60. 10.4049/jimmunol.1402409 25911752 PMC4433824

[52] Ong S-M Hadadi E Dang T-M Yeap W-H Tan CT-Y Ng T-P. The pro-inflammatory phenotype of the human non-classical monocyte subset is attributed to senescence. Cell Death Dis. (2018) 9:266. 10.1038/s41419-018-0327-1 29449647 PMC5833376

[53] Misharin AV Cuda CM Saber R Turner JD Gierut AK Haines GK. Nonclassical Ly6C(-) monocytes drive the development of inflammatory arthritis in mice. Cell Rep. (2014) 9:591–604. 10.1016/j.celrep.2014.09.032 25373902 PMC4223808

[54] Cros J Cagnard N Woollard K Patey N Zhang SY Senechal B. Human CD14(dim) monocytes patrol and sense nucleic acids and viruses *via* TLR7 and TLR8 receptors. Immunity. (2010) 33:375–86. 10.1016/j.immuni.2010.08.012 20832340 PMC3063338

[55] Nazareth N Magro F Silva J Duro M Gracio D Coelho R. Infliximab therapy increases the frequency of circulating CD16(+) monocytes and modifies macrophage cytokine response to bacterial infection. Clin Exp Immunol. (2014) 177:703–11. 10.1111/cei.12375 24816497 PMC4137855

[56] Aeberli D Kamgang R Balani D Hofstetter W Villiger PM. Regulation of peripheral classical and non-classical monocytes on infliximab treatment in patients with rheumatoid arthritis and ankylosing spondylitis. Rmd Open. (2016) 2:e000079. 10.1136/rmdopen-2015-000079 26819749 PMC4716562

[57] Geissmann F Jung S Littman DR. Blood monocytes consist of two principal subsets with distinct migratory properties. Immunity. (2003) 19:71–82. 10.1016/S1074-7613(03)00174-2 12871640

[58] Soehnlein O Steffens S Hidalgo A Weber C. Neutrophils as protagonists and targets in chronic inflammation. Nat Rev Immunol. (2017) 17:248–61. 10.1038/nri.2017.10 28287106

[59] Ohmori M Kitoh Y Harada K Sugimoto K. Polymorphonuclear leukocytes (PMNs) functions in SHR, L-NAME- and DOCA/salt-induced hypertensive rats. J Hypertens. (2000) 18:703–7. 10.1097/00004872-200018060-00007 10872554

[60] Chatterjee M Saluja R Tewari S Barthwal MK Goel SK. Augmented nitric oxide generation in neutrophils: Oxidative and pro-inflammatory implications in hypertension. Free Radic Res. (2009) 43:1195–204. 10.3109/10715760903247256 19905982

